# Genome3D: exploiting structure to help users understand their sequences

**DOI:** 10.1093/nar/gku973

**Published:** 2014-10-27

**Authors:** Tony E. Lewis, Ian Sillitoe, Antonina Andreeva, Tom L. Blundell, Daniel W.A. Buchan, Cyrus Chothia, Domenico Cozzetto, José M. Dana, Ioannis Filippis, Julian Gough, David T. Jones, Lawrence A. Kelley, Gerard J. Kleywegt, Federico Minneci, Jaina Mistry, Alexey G. Murzin, Bernardo Ochoa-Montaño, Matt E. Oates, Marco Punta, Owen J.L. Rackham, Jonathan Stahlhacke, Michael J.E. Sternberg, Sameer Velankar, Christine Orengo

**Affiliations:** 1 Institute of Structural and Molecular Biology, UCL, 636 Darwin Building, Gower Street, London, WC1E 6BT, UK; 2 MRC Laboratory of Molecular Biology, Hills Road, Cambridge, CB2 0QH, UK; 3 Department of Biochemistry, University of Cambridge, Old Addenbrooke's Site, 80 Tennis Court Road, Cambridge, CB2 1GA, UK; 4 Department of Computer Science, UCL, Gower Street, London, WC1E 6BT, UK; 5 European Bioinformatics Institute, Wellcome Trust Genome Campus, Hinxton, Cambridgeshire, CB10 1SD, UK; 6 Centre for Bioinformatics, Department of Life Sciences, Imperial College London, London, SW7 2AZ, UK; 7 Department of Computer Science, University of Bristol, Merchant Venturers Building, Woodland Road, Bristol, BS8 1UB, UK; 8 MRC Clinical Sciences Centre, Hammersmith Hospital Campus, Du Cane Road, London, W12 0NN, UK

## Abstract

Genome3D (http://www.genome3d.eu) is a collaborative resource that provides predicted domain annotations and structural models for key sequences. Since introducing Genome3D in a previous NAR paper, we have substantially extended and improved the resource. We have annotated representatives from Pfam families to improve coverage of diverse sequences and added a fast sequence search to the website to allow users to find Genome3D-annotated sequences similar to their own. We have improved and extended the Genome3D data, enlarging the source data set from three model organisms to 10, and adding VIVACE, a resource new to Genome3D. We have analysed and updated Genome3D's SCOP/CATH mapping. Finally, we have improved the superposition tools, which now give users a more powerful interface for investigating similarities and differences between structural models.

## INTRODUCTION

Though solved structures are now vastly outnumbered by sequences, they still offer insights that are invaluable in attempts to understand the sequence data and implications to functional mechanisms. Genome3D (1) applies the combined expertise of six leading UK-based structural bioinformatics groups (Blundell, Gough, Jones, Murzin, Orengo and Sternberg) to bring new understanding to sequences and to determine where a consensus can be reached. The resources involved in Genome3D (DomSerf (2), FUGUE (3), Gene3D (4), pDomTHREADER (5), PHYRE2 (6), SUPERFAMILY (7), VIVACE, CATH (8), SCOP (9)) are summarized in Table 1.

**Table 1.** Summary of the Genome3D prediction resources and the structural domain classifications, on which they are based

Resource	Principal investigator	Affiliation	Type	Classification source
DomSerf (2)	Jones	UCL	Prediction: Models	CATH
FUGUE (3)	Blundell	Cambridge	Prediction: Annotations	CATH/SCOP
Gene3D (4)	Orengo	UCL	Prediction: Annotations	CATH
pDomTHREADER (5)	Jones	UCL	Prediction: Annotations	CATH
PHYRE2 (6)	Sternberg/Kelley	Imperial	Prediction: Both	SCOP/PDB
SUPERFAMILY (7)	Gough	Bristol	Prediction: Both	SCOP
VIVACE	Blundell	Cambridge	Prediction: Models	CATH/SCOP
CATH (8)	Orengo	UCL	Classification	N/A
SCOP (9)	Murzin	MRC-LMB	Classification	N/A

Our resources are used to annotate sequences from 10-model organisms with predicted domain annotations and 3D structural models. These predictions are based on the structural domains in the SCOP and CATH databases. By providing multiple predictions for the same sequence, we allow our users to assess the degree of consensus between the resources and hence gauge confidence accordingly.

Genome3D also contains a mapping between the SCOP and CATH databases, which allows us to identify pairs of SCOP/CATH superfamilies that can be considered equivalent. We exploit this on the website to highlight where predictions assign domains to equivalent SCOP/CATH superfamilies.

## IMPROVEMENTS

### Improved coverage of sequence space using Pfam representatives

During the past 2 years, we focused effort on increasing Genome3D's coverage of sequence space. Since structural prediction methods can be computationally intensive, it was important to cover sequence space efficiently. So our aim was to choose a moderate set of sequences for annotation that would represent as much sequence space as possible.

To choose this set, we used Pfam which has ‘sequence coverage of the UniProt Knowledgebase (UniProtKB) at nearly 80%’ (10). In particular, we used Pfam version 27.0, which was released in March 2013, was live at the time of writing and contains 14 831 Pfam families.

Pfam families represent domains, whereas Genome3D annotates whole UniProt sequences. Fortuitously, 14 527 (98.0%) of the families had at least one sequence that contained nothing other than a single copy of the family's domain. Only 304 families (2.0%) had no such sequence. Another 6474 families (43.7%) already had members that were annotated in Genome3D and another 262 families (1.8%) only contained UniProt fragments.

This left 7791 families (52.5%) from which to choose a representative to be annotated in Genome3D. We collaborated with Pfam to discuss how to choose sensible representatives. They highlighted the issue of a *Pfam fragment*, a domain match that is less than 95% of the true length of the family's domain. We removed these Pfam fragments where possible, then chose the sequence with the highest HMMER (11) bit score to the family (using data provided by Pfam). In 562 (7.2%) of the 7791 cases, we were forced to choose a Pfam fragment and so chose the one with the longest match length.

Two of the Genome3D domain annotation methods (Gene3D and SUPERFAMILY) use fast, sequence-based approaches so they are well suited to handling larger data sets and longer, multi-domain sequences. To exploit this, we selected an additional 24 825 UniProt sequences as representatives of the multi-domain *architectures* (layouts of family domains on their full protein sequences) described by Pfam.

### Sequence-based search

As described above, Genome3D focuses on good coverage of representative sequences. This makes it important that Genome3D helps users to find Genome3D-annotated sequences that are most similar to their sequences of interest.

With this in mind, we have implemented a sequence search based on jackhmmer (11), and ensured that the keyword and sequence searches feature prominently on the home page. The sequence search results are typically returned within a few seconds and are displayed in ascending order of jackhmmer *E*-value. All search results can be filtered by species.

### Coverage and new structural model method ‘VIVACE’

Since the previous paper, the Genome3D groups have extended and improved their Genome3D data. In particular, the Blundell group has contributed structural models from VIVACE, a method new to Genome3D.

The VIVACE pipeline makes use of the domains identified by FUGUE to select a set of at most five of the best templates from the TOCCATA database according to various criteria, including: similarity to the query and to each other, coverage of the target sequence, crystallographic quality and conformational compatibility. The selected templates are aligned to each other using BATON and then FUGUE is used to incorporate the query into it. This alignment is fed into MODELLER to generate a model, which is then rated by a battery of quality assessment programs to analyse its reliability. The resource URL is http://structure.bioc.cam.ac.uk/toccata.

The addition of VIVACE brings the number of structural model methods to four. Of the 20 196 sequences in the human data set, 9472 (46.9%) are now annotated by all four methods; 17 796 (88.1%) by at least one. There are six domain annotation methods. Of the 20 196 sequences in the human data set, 11 723 (58.0%) are now annotated by all six methods; 19 096 (94.6%) by at least one.

**Figure 1.**
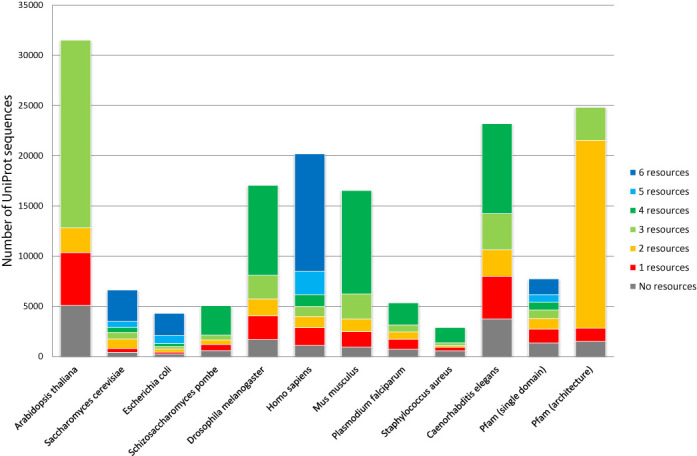
Coverage of UniProt sequences in each genome by the number of resources providing at least one **domain annotation** for the sequence. ‘Pfam (single domain)’ denotes the new data set of sequences representing Pfam families and ‘Pfam (architecture)’ denotes the extra data set of sequences representing the Pfam multi-domain architectures, as discussed in the main text.

**Figure 2.**
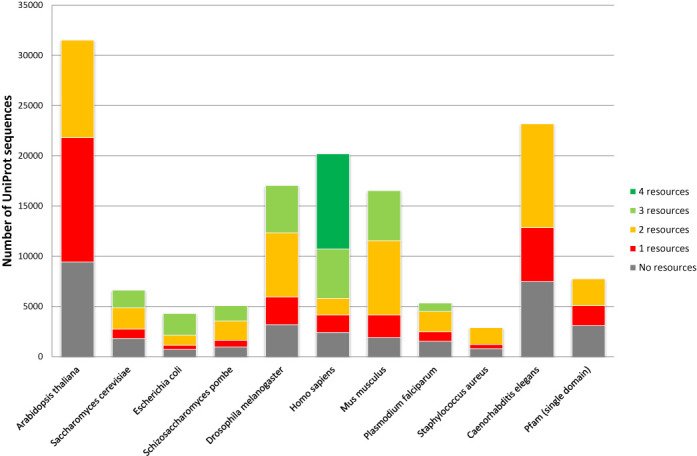
Coverage of UniProt sequences in each genome by the number of resources providing at least one **structural model** for the sequence. ‘Pfam (single domain)’ denotes the new data set of sequences representing Pfam families, as discussed in the main text.

Figures 1 and 2 show Genome3D's coverage of the 10 genomes and of the 2 new Pfam sets (described above).

### Updates to the SCOP/CATH mapping

Genome3D includes a mapping between SCOP and CATH, which we generate by calculating all overlaps between all domains in the two resources and then aggregating these to compare superfamilies. We use this mapping to identify *consensus superfamily pairs*—pairs of superfamilies (one from SCOP; one from CATH) that are more similar to each other than to any other. These consensus superfamily pairs are categorized into bronze, silver and gold standard to indicate the level of similarity between their two superfamilies.

Since the previous paper, we have developed new internal pages for curating the relationships between SCOP and CATH. These pages have permitted an analysis of the mapping that has revealed that some of the previous criteria for the gold standard were needlessly strict. The previous gold criteria demanded not only that each superfamily have at least 80% of their domains mapped to the other superfamily, but also that this was true when penalizing for differences in domains not yet classified. This extra requirement has now been dropped and the change has upgraded 393 consensus superfamily pairs from silver standard to gold. The current numbers for the mapping between SCOP v1.75 and CATH v3.5.0 are: 763 gold pairs, 134 silver pairs and 532 bronze pairs. The criteria are now as described below.

Bronze Pairs:

- are more similar to each other than to any other superfamily.

Silver Pairs:

- meet the Bronze criteria,
- have at least 80% of each superfamily's domains mapping to the other (ignoring differences in domains not yet classified) and
- contain domains in each superfamily that map to domains in the other over an average of at least 80% of their residues.

Gold Pairs:

- meet the Silver criteria and
- contain domains in each superfamily that map to domains in the other over a minimum of at least 80% of their residues.

### Static images of superpositions

The previous paper described how the Genome3D web pages allow users to select overlapping structural models and then view a superposition of those structural models. At that time, the superpositions could be viewed in PyMOL or Jmol. We have now added an interface that dynamically renders a static image of the superposition so it can be viewed on almost any graphical browser.

The interface provides buttons that allow the user to rotate the superposed structures and to specify how they should be coloured. Each click on these buttons triggers an update of the static image.

### Superposition algorithm improvements

The superpositions are implemented through dynamic requests to the webserver, which runs a fast superposition algorithm and returns the results in the specified format. To perform the superposition, the algorithm must first build an alignment of equivalent residues. Since the structures being superposed are predicted models of overlapping regions of the same sequence, they are aligned based on residue number. This is simpler and faster than aligning based on sequence or structure and also better represents the meaning of the superposition.

**Figure 3.**
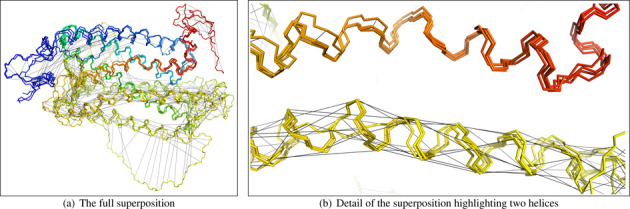
PyMOL downloads of Genome3D superpositions now include black alignment lines that connect the locations in which the different structural models place the same residue from the source sequence. These images show a superposition of four resources’ structural models of OR5AL1 (UniProt accession: P0C617) with the alignment lines displayed. In (b), the models predict two structurally similar helices but differ about where to locate the source sequence's residues on the yellow helix at the bottom.

Since the last paper, we make several improvements to this superposition algorithm:

- The algorithm now disregards the most divergent positions when superposing the structures so that divergent regions do not disrupt the superposition of similar regions. This is most notable in extreme cases, such as those including models with long, unfolded tails, but it also tightens superpositions for more similar sets of models. The divergent regions are identified using a scoring approach based on that used in the SSAP algorithm (12).
- The algorithm now offers the option to colour the superposition with a rainbow gradient (blue through to red for N-terminus through to C-terminus). Since the algorithm knows the alignment of the structures, it can do better than just separately rainbow-colouring each structure; it can uniformly paint the full stretch of modelled sequence. This means that each residue in the original sequence is assigned one colour, which is used to paint that residue in all models. This makes it visually obvious which parts of the superposed structures are modelling identical parts of the source sequence. The technique is particularly powerful when superposing a patchwork of overlapping regions.
  Further, the scores that are used to identify divergent regions are also used to determine the rate of change of the rainbow gradient: the colour changes slowly over divergent regions so that the majority of the colour spectrum is reserved for better conserved regions.
  At present, this rainbow colouring can be applied to the static images and the PyMOL downloads.
- The algorithm now adds information about the alignment to the PyMOL download. This enables PyMOL to display black lines that connect equivalent residues in the superposed structures. This can be activated by clicking on the ‘alignment’ button at the right of the PyMOL window. To reduce clutter, each position in the alignment is represented by a minimal spanning tree between the equivalent residues’ coordinates. These alignment lines can be a powerful tool for examining differences between models. For example, Figure 3 illustrates how the lines reveal differences between four resources’ models for OR5AL1 (UniProt accession: P0C617). Though the models share structural similarity and are in strong agreement for the red helix at the top, the alignment lines reveal that they differ about where to assign residues from the source sequence on the yellow helix at the bottom.

### Other improvements and updates

We have made many other minor improvements and updates to Genome3D since the previous paper, such as refining the source sequence data set and adding a link to automatically submit the source sequence to a group's resource. We have also developed a substantial tutorials section of the website, which is accessible by clicking ‘Tutorials’ on the navigation bar at the top of all Genome3D pages. This provides both general Genome3D tutorials (search; exploring structural models) and specific resource tutorials that explore how the resources’ features can help users to uncover more information about their sequences.

## CONCLUSIONS

Genome3D is a collaborative resource that provides structure-based predictions to help users learn more about their sequences. By combining results from independent resources, it allows the user to assess agreement and hence gauge confidence.

We have improved Genome3D substantially since introducing it in a previous NAR paper. We have increased coverage by annotating at least one representative from of as many Pfam families as possible. We have provided users with a fast sequence search. We have updated and extended the data, enlarging the source data set from 3 genomes to 10 and adding VIVACE, a method new to Genome3D.6 We have updated the criteria that are used for the mapping between SCOP and CATH. We have improved the website, not least by adding dynamically generated images of superpositions. We have improved the algorithm that generates the superpositions to make it more useful for exploring similarities and differences between models. We have made many other smaller improvements, such as adding a substantial set of tutorials to the website.

These updates strengthen Genome3D as a resource for exploring the insights that structure can bring to sequence and as a gateway for then learning more through the groups’ individual resources.
